# Expression profile of long noncoding RNAs in post-stroke aphasia

**DOI:** 10.3389/fneur.2025.1504028

**Published:** 2025-09-15

**Authors:** Linazi Gu, Caili Ren, Mei Qu, Hui Chang, Yanling Xi

**Affiliations:** ^1^Department of Rehabilitation Medicine, The First Affiliated Hospital of Xinjiang Medical University, Ürümqi, China; ^2^Department of Rehabilitation Medicine, Wuxi Central Rehabilitation Hospital, Mental Health Center Affiliated to Jiangnan University, Wuxi, China; ^3^Department of Rehabilitation Medicine, Shanghai Pudong New Area Guangming Hospital of Traditional Chinese Medicine, Shanghai, China; ^4^School of Foreign Languages, Shanghai Jiao Tong University, Shanghai, China

**Keywords:** post-stroke aphasia, long noncoding RNA, RNA sequencing, RP11-227G15.3, biomarker

## Abstract

**Objective:**

Post-stroke aphasia (PSA) is one of the most common complications after stroke, seriously affecting survivors' quality of life. While long noncoding RNAs (lncRNAs) are linked to stroke, their role in PSA remains unclear. This study explored PSA-associated lncRNA expression to identify potential biomarkers.

**Methods:**

LncRNA expression profiles were analyzed via high-throughput RNA sequencing (RNA-seq) with subsequent quantitative polymerase chain reaction (qPCR) validation. Gene Ontology (GO), Kyoto Encyclopedia of Genes and Genomes (KEGG) enrichment, and correlations with language behaviors were assessed.

**Results:**

Initial analyses comparing PSA and stroke patients revealed 797 significantly differentially expressed lncRNAs (69 upregulated, 728 downregulated), linked to several metabolic pathways. qPCR confirmed upregulation of lncRNA RP11-227G15.3 in PSA. A negative correlation with oral spelling scores was observed in 12 PSA patients (*p* = 0.04), but this did not survive Bonferroni correction, with no significance for other language measures.

**Conclusion:**

This is an exploratory pilot study. LncRNA RP11-227G15.3 represents a candidate biomarker requiring validation for PSA, though its preliminary association with oral spelling scores requires validation in larger, independent cohorts before any clinical application.

## Introduction

Post-stroke aphasia (PSA) is a severe stroke complication characterized by the impairment of the brain's language control center as a consequence of stroke-related vascular or nerve damage in this region (1). PSA patients commonly experience communication difficulties (2), adversely impacting patient quality of life and contributing to higher levels of social isolation and depression (3). These negative outcomes, in turn, place a greater burden on the patient's family and community, emphasizing the need to establish reliable biomarkers associated with PSA given the importance of this medical issue.

Ischemic stroke often leads to post-stroke aphasia (PSA), a common complication affecting language function (4), but its underlying molecular mechanisms remain poorly understood. Long noncoding RNAs (lncRNAs) are > 200 nucleotides long and generally lack coding potential (5), have emerged as key regulators in various diseases, exerting effects not through translation but by modulating signaling pathways and protein-coding genes (6). With advances in sequencing technologies, accumulating evidence has linked lncRNA dysregulation to IS, for example, altered lncRNA expression profiles in peripheral blood mononuclear cells (PBMCs) of IS patients (7), dynamic changes in PBMC gene expression during acute IS (8), and specific IS-associated lncRNAs such as H19, MALAT1, and XIST (9–11). However, research on lncRNAs in PSA remains scarce—most lncRNAs related to PSA have not been identified or characterized, and their potential roles in PSA pathogenesis are unclear.

Given that peripheral blood mononuclear cells (PBMCs) serve as accessible proxies for systemic molecular changes, we aimed to fill this gap by performing high-throughput RNA-sequencing (RNA-seq) to profile lncRNA expression in PBMCs from stroke and PSA samples. The study seeks to identify differentially expressed lncRNAs (DElncRNAs) associated with PSA, explore their potential functional roles through pathway enrichment analyses, and lay a foundation for understanding PSA mechanisms and developing novel biomarkers.

## Materials and methods

### PBMC isolation

For this study, 29 patients were recruited from whom peripheral blood samples were collected, including 12 PSA patients, these same PSA patients following treatment (PSA-T), 9 stroke patients, and 8 healthy control (HC) subjects. Patients were strictly enrolled with stroke confirmed by computerized tomography/magnetic resonance imaging. Lesion laterality was recorded as left hemisphere right hemisphere or bilateral. Aphasia was diagnosed using the Chinese Aphasia Battery (PSA: < 80; stroke: ≥95). Stroke severity was assessed by the National Institutes of Health Stroke Scale (NIHSS) at admission, with data collected from clinical records. Characteristic features of the study population are listed in Table 1. Blood samples were collected from PSA, stroke, and HC groups at the time of hospital admission, while the PSA-T samples were collected at the time of discharge. PBMCs from these different patient blood samples were separated via Ficoll density gradient centrifugation based on provided instructions, and PBMCs were stored at −80 °C for further analysis.

**Table 1:** Characteristic features of study cohort.

**Characteristics**	**HC**	**Stroke**	**PSA (PSA-T)**
Number	8	9	12
Gender (Male/Female)	3/5	5/4	7/5
Age (years)	60.75 ± 7.59	59.56 ± 8.40	61.00 ± 13.07
Disease duration (days)	–	14.89 ± 2.57	52.67 ± 28.14^*^
Cerebrovascular disease	–	Cerebral hemorrhage (1) Cerebral infarction (8)	Cerebral hemorrhage (1) Cerebral infarction (11)
Lesion laterality	–	Right hemisphere (9)	Left hemisphere (9) Bilateral (2)
NIHSS score	–	5.89 ± 1.56	15.58 ± 4.82^*^

HC, healthy control; PSA, Post-stroke aphasia; PSA-T, PSA patients following treatment. Statistically significant parameter (*p* < 0.05) between PSA and Stroke patients is marked with an asterisk (^*^).

The Shanghai Pudong New Area Guangming Hospital of Traditional Chinese Medicine Ethics Committee provided approval for this study, which was conducted as per the Declaration of Helsinki. Written informed consent was obtained from all patients.

### RNA isolation

TRIzol (15596018, Invitrogen, MA, USA) was used to extract RNA from patient PBMCs based on provided directions, after which a NanoDrop 2000 spectrophotometer (Thermo Fisher Scientific, MA, USA) was used to quantify RNA levels in individual samples. The integrity and purity of RNA samples were assessed through 1% agarose gel electrophoresis, with further Agilent 2100 Bioanalyzer (Agilent Technologies, CA, USA) analyses to confirm the quality and integrity of the RNA. The prepared RNA samples were then stored at −80 °C.

### RNA-Seq library construction

After using the Ribo-zero rRNA removal kit (Epicentre, WI, USA) to remove rRNA, ethanol precipitation was used to clean the processed RNA, and an Ultra II RNA Library Prep Kit for Illumina (New England Biolabs) was used as directed to prepare cDNA libraries. These libraries were, in turn, purified and assessed using an Agilent Bioanalyzer 2100 instrument (Agilent Technologies). Paired-end sequencing of these samples was then performed by Wuhan Ruixing Biotechnology Co. Ltd (Wuhan, China) using a NovaSeq Xplus instrument (Illumina Novaseq, CA, USA).

To obtain clean reads, adapters, low-quality reads, and poly-N reads were removed. Clean read quality was then evaluated based on Q20, Q30, and GC content values, after which these data were aligned with the human genome (GRCh38. p13 database, NCBI) with HISAT2. Raw sequencing data were filtered to remove low-quality reads, yielding 122.8 ± 71.8 million clean reads per sample with Q30 base ratio > 93%, GC content = 48% (Supplementary File S1). For alignment metrics, the average mapping rate is 96.03 ± 0.59%, with a unique mapping rate of ≥ 90%.

### lncRNA identification and differential expression analyses

After grouping the RNA-seq data, individual groups were assembled and StringTie was used to predict transcripts. During the lncRNA prediction phase, transcripts with FPKM < 1 were first filtered out to reduce noise from low-abundance artifacts. Subsequent multi-layer screening was applied to refine putative lncRNAs: (1) extracting transcripts with class_code “uxi”; (2) excluding those with coding potential via four independent tools (Coding Potential Calculator-CPC2, Coding-Non-Coding Index-CNCI, Coding Potential Assessment Tool-CPAT, and ORF Length and GC content-LGC); (3) removing transcripts < 1,000 bp from the nearest known gene; (4) retaining multi-exon transcripts ≥ 200 bp and single-exon transcripts ≥ 1,000 bp. Filtered transcripts were merged using StringTie, and putative lncRNAs were further annotated by comparison with the non-coding RNA database NONCODEv6 (http://www.noncode.org/), with known lncRNAs referenced against genome annotations and novel lncRNAs (e.g., XLOC loci) validated by both computational non-coding evidence and structural criteria. Details were included in Supplementary File S2.

For differential expression analysis, raw read counts (not normalized values) were supplied to DESeq2, which models raw counts and uses scaling factors to account for library depth differences. DElncRNAs were identified by pairwise sample comparisons, defined by linear fold change (FC) ≥ 1.5 (upregulated) or ≤ 0.67 (downregulated) and unadjusted *p* value < 0.01.

### Functional enrichment analyses

Cis-target analyses were conducted to identify targets of DElncRNAs. For this analysis, protein-coding genes within 100 kb upstream or downstream of these lncRNAs were established as cis-target genes. The DAVID (https://david.ncifcrf.gov/) was used to perform Gene Ontology (GO) and Kyoto Encyclopedia of Genes and Genomes (KEGG) pathway enrichment analyses of these targets to better clarify the functional roles of these lncRNAs. GO enrichment analyses included the annotation of biological process, molecular function, and cellular component terms. KEGG analyses were similarly used to identify pathways related to these DElncRNAs. Term enrichment was determined by false discovery rates (FDR) < 0.05.

### qPCR validation

To validate differential expression from RNAseq data, 6 DElncRNAs (selected from the candidates meeting |Log2 FC| > 1.45, FDR < 0.05, and baseMean ≥ 10) were subjected to qPCR analysis. The four lncRNAs that differed most markedly in this first analysis were then used for qPCR validation incorporating the PSA-T and HC group samples. After extracting RNA from PBMCs with TRIzol as above, a reverse transcription kit (R323-01, Vazyme, Nanjing, China) was used to prepare cDNA with a MyCycler (T100, Bio-Rad, CA, USA) and the following settings: 42 °C for 5 min, 37 °C for 15 min, 85 °C for 5 s. Next, qPCR analyses were performed using an ABI QuantStudio 5 instrument with the following settings: 95 °C for 10 min; 40 cycles of 95 °C for 15 s and 60 °C for 1 min. Each 10 μL reaction consisted of 5 μL of Hieff qPCR SYBR Green Master Mix (11202ES03, Yeasen, Shanghai, China), 0.5 μL each of the forward (F) and reverse (R) primers at 10 μM, 2 μL of cDNA, and 2 μL of nuclease-free water. Three technical replicates were run per sample, and transcript concentrations were normalized to GAPDH, using the 2^−ΔΔCT^ method for relative expression analyses. Utilized primers were as follows:

RP11-69H7.3-F: GAGAACACACATGGGCTTTGG

RP11-69H7.3-R: GGCTCAGCAGAGGTGAAGTT

RP11-104L21.2-F: CCTGTGTTCGACTCATCTTACG

RP11-104L21.2-R: TTGCTGAAAGTAGCGCAGTTT

XLOC_000019-F: GGCGCAATGAAGGTGAAGG

XLOC_000019-R: CCATCTTTCGGGTCCTAACAC

XLOC_000018-F: GGCGCAATGAAGGTGAAGG

XLOC_000018-R: CATCTTTCGGGTCCTAACACG

RP11-732A19.10-F: CATTTAGGTTGGGTGGCTCAG

RP11-732A19.10-R: ACCGCAAGTACACTCTAACCA

RP11-227G15.3-F: CAATAGATGCCGTACCAAATGC

RP11-227G15.3-R: TCTGTTCAAGAAGTCGTCGTC

GAPDH-F: GGTCGGAGTCAACGGATTTG

GAPDH-R: GGAAGATGGTGATGGGATTTC

### Language behavioral evaluation

The standardized Aphasia Battery in Chinese tool, which is a revised version of the Western Aphasia Battery that has been adapted for use based on Chinese culture, is the most widely used tool to assess aphasia in China, and it has been established to be valid and reliable. For this study, a single professional language therapist used this tool to assess the language function of enrolled patients, assessing only oral expression (information content, fluency, retelling, and naming ability), reading (visual reading, calligraphy and painting matching, reading instruction execution, listening recognition, and blank filling), writing (surname and address, dictation, transcription, picture writing, serial writing, and spontaneous writing), listening comprehension (whether to answer questions, listening recognition, and oral instructions), utilization, structure, visuospatial, and calculation tasks.

### Statistical analyses

Correlation analyses were performed to explore the associations between levels of lncRNA expression and patient language behavior scores. Spearman correlation coefficients were calculated based on data distribution, and Bonferroni correction was applied for multiple testing. For confounding factor adjustment including disease duration, lesion laterality and NIHSS score, multivariate linear regression models were constructed. To assess differences in lesion laterality between groups, the Chi-square test was employed. GraphPad Prism (v9.1.2, GraphPad Software, CA, USA) and SPSS (v11.0, IBM, IL, USA) were used to perform all analyses, presenting the results as means ± standard deviation.

## Results

### PSA-associated lncRNA profiling

**Figure 1:**
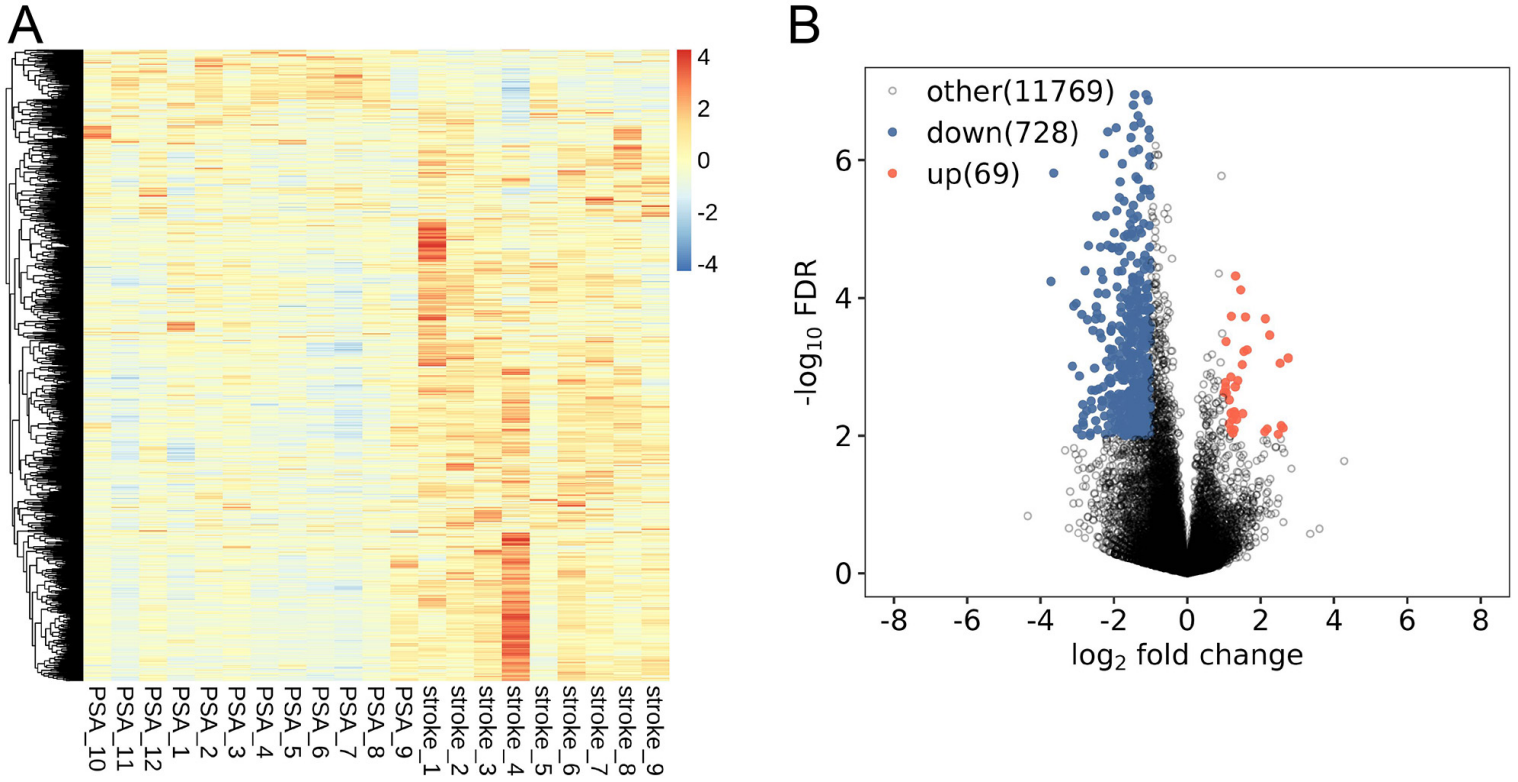
PSA-related lncRNA expression profiling. **(A)** lncRNAs differentially expressed between post-stroke aphasia (PSA) and stroke patients. **(B)** A volcano plot of differential lncRNA expression. Red and blue respectively denote upregulated and downregulated lncRNAs.

To clarify PSA-related patterns of lncRNA expression, PBMCs from 12 PSA patients and 9 stroke patients were used to perform high-throughput RNA sequencing. Lesion laterality differed significantly between groups with all stroke patients having right hemisphere lesions and most PSA patients having left hemisphere lesions 9 cases with 2 cases having bilateral lesions (*p* < 0.05). NIHSS scores were higher in the PSA group than in the stroke group (*p* < 0.05, Table 1). The 16,888 annotated lncRNAs were displayed with hierarchically clustered heatmaps (Figure 1A), revealing clear expression differences among groups. These results were also visualized with a volcano plot (Figure 1B). Relative to the stroke group, 797 DElncRNAs (69 upregulated, 728 downregulated) were identified in the PSA group (Supplementary File S3).

### Functional enrichment analyses of DElncRNAs co-expression with all expressed genes through cis-regulation

**Figure 2:**
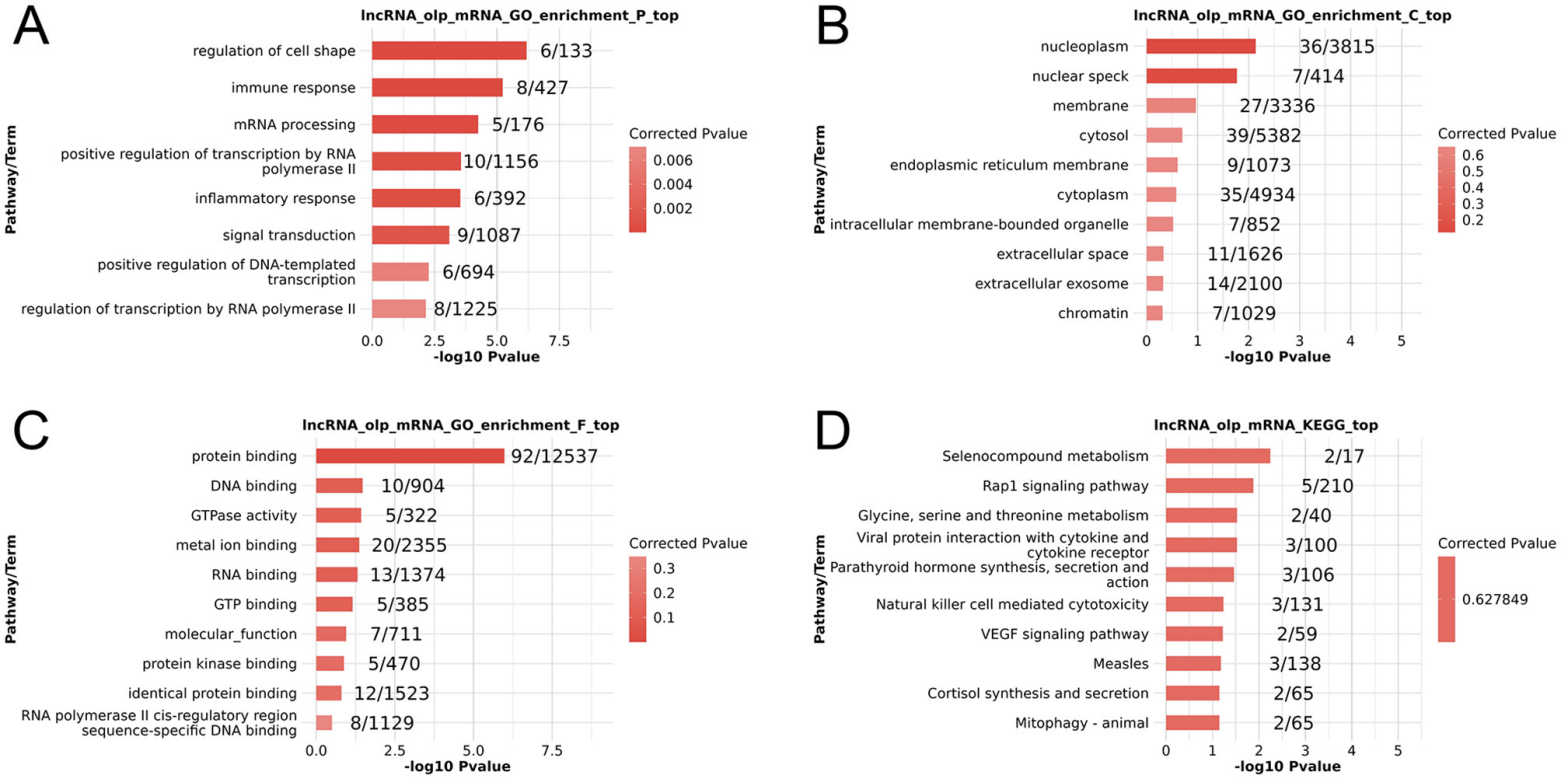
Functional enrichment analyses of PSA-associated differentially expressed lncRNAs. **(A–C)** GO Biological process **(A)**, cellular component **(B)**, and molecular function **(C)** enrichment results. **(D)** KEGG pathway enrichment results. The enrichment terms presented are based on unadjusted *p*-values.

In an effort to begin exploring the functional roles that these DElncRNAs may play in the context of PSA, GO and KEGG enrichment analyses of their cis-target genes were conducted (Supplementary File S4). The top 10 enriched GO terms in each category were graphed (Figures 2A–C), revealing potential enrichment in terms associated with the regulation of cell shape and immune responses, however, these enrichment results are based on unadjusted *p*-values without multiple-testing correction, and these enrichments reflecting *in silico* functional annotations of cis-target genes derived from PBMCs rather than direct evidence for processes in the central nervous system (CNS). Similarly, the top 10 enriched KEGG pathways were graphed (Figure 2D), with selenocompound metabolism and Rapl signaling pathways appearing among the most enriched.

### qPCR-based validation of DElncRNAs

To refine the pool of DElncRNAs, 797 initially identified DElncRNAs were further filtered using stricter criteria: |Log2 FC| > 1.45, FDR < 0.05, and baseMean ≥ 10, resulting in 128 high-confidence DElncRNAs (Supplementary File S3). Statistical power analysis confirmed that the large effect size threshold (|log_2_FC|>1.45) ensured >95% power to detect true differences, while 1,000 permutation tests estimated an expected FDR of ~7.8% (8–12 false positives among 128 significant genes), supporting the reliability of the refined set.

**Figure 3:**
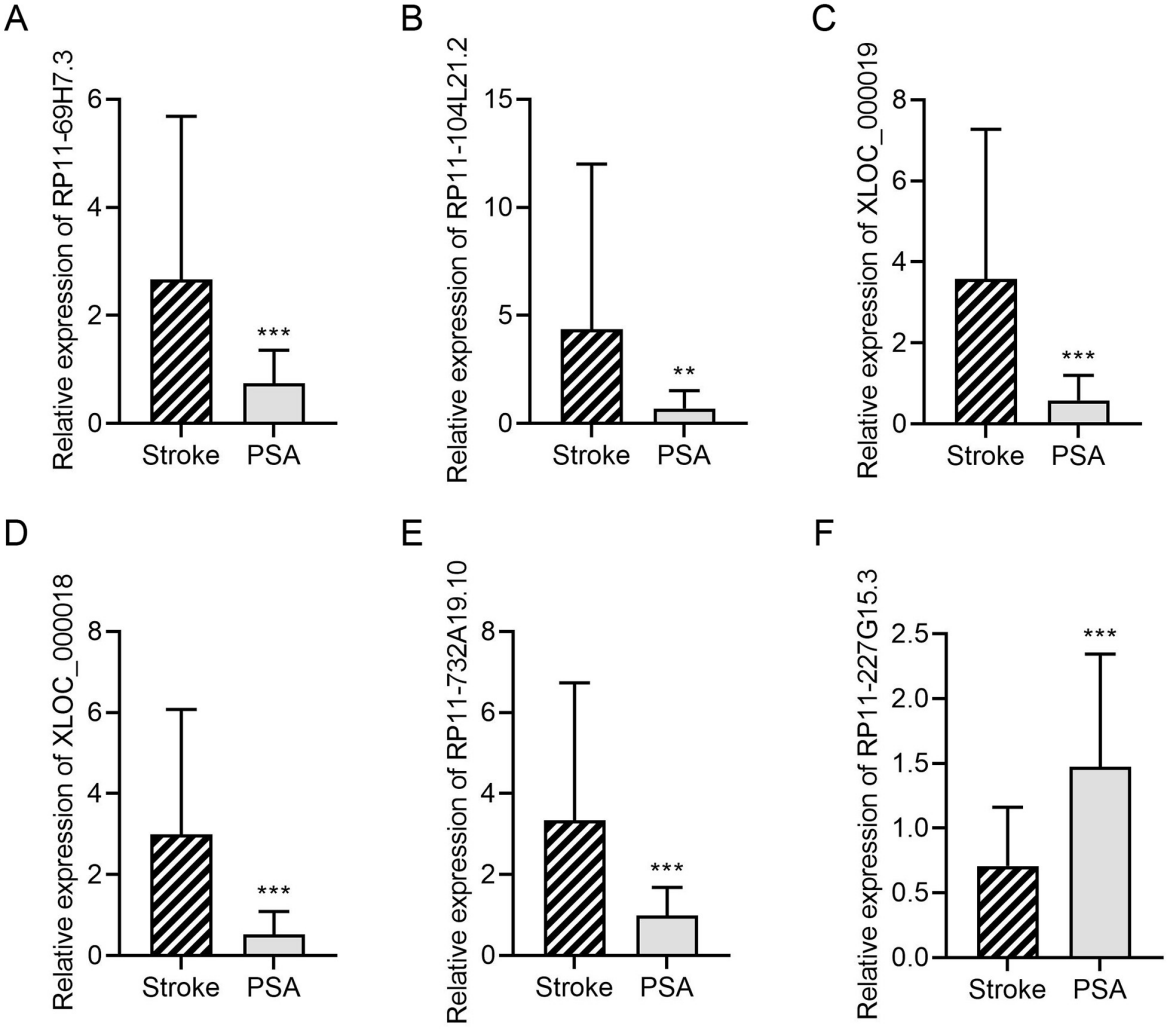
DElncRNA validation by qPCR. A qPCR approach was used to validate the relative expression of **(A)** RP11-69H7.3; **(B)** RP11-104L21.2; **(C)** XLOC_000019; **(D)** XLOC_000018; **(E)** RP11-732A19.10; **(F)** RP11-227G15.3. GAPDH was utilized for normalization (**P < 0.01, ***P < 0.001 vs. stroke).

**Figure 4:**
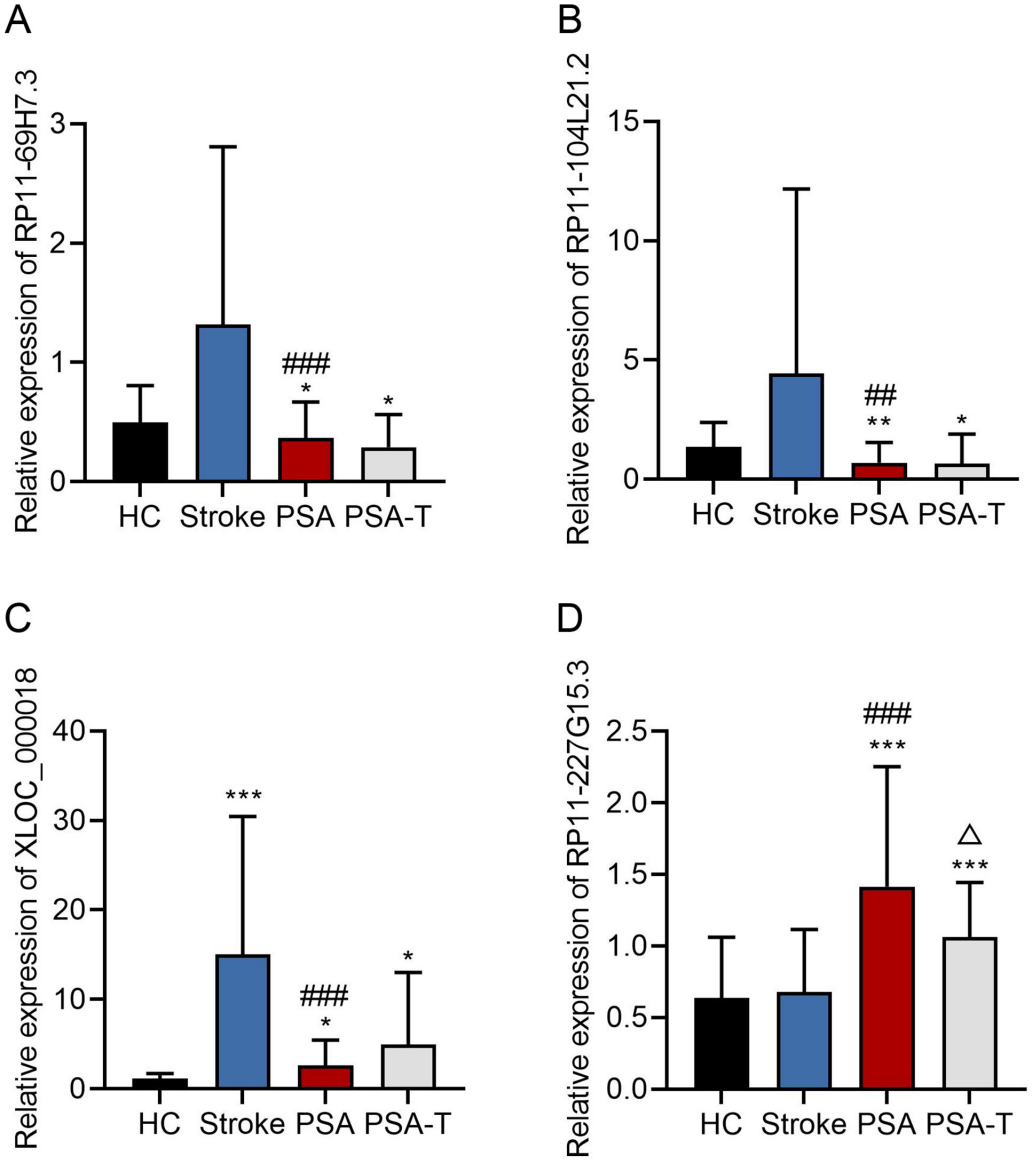
qPCR analyses of lncRNA expression. A qPCR approach was used to assess the relative expression levels of **(A)** RP11-69H7.3; **(B)** RP11-104L21.2; **(C)** XLOC_000018; **(D)** RP11-227G15.3. GAPDH was used for normalization (*P < 0.05, **P < 0.01, ***P < 0.001 vs. HC; ^##^*P* < 0.01, ^###^*P* < 0.001 vs. stroke; Δ < 0.05 vs. PSA). The enrichment terms related to these lncRNAs (as referenced in functional analyses) are unadjusted.

For validation, 6 lncRNAs from this 128-gene set were selected for qPCR analysis, including 4 significantly downregulated (RP11-69H7.3, RP11-104L21.2, XLOC_000019, XLOC_000018) and 2 significantly upregulated (RP11-227G15.3, RP11-732A19.10) in stroke and PSA patients. Of these, 5 (83%) showed consistent expression trends with RNA-seq data (Figure 3). The top four lncRNAs that were most differentially expressed were further evaluated to compare their expression in the stroke, PSA, PSA-T, and HC patient groups (Figure 4). The qPCR analysis revealed that RP11-69H7.3, RP11-104L21.2, and XLOC_000018 were significantly downregulated in both PSA and PSA-T patients compared with the stroke group (based on unadjusted *p*-values for pairwise comparisons). RP11-227G15.3 was significantly upregulated in PSA patients but downregulated in the PSA-T group, under the same statistical conditions. While RP11-227G15.3 appears to differentiate among PSA, PSA-T, and stroke patients, these findings are exploratory due to the use of unadjusted *p-*values, and its status as a candidate biomarker requiring validation needs confirmation in larger, independent cohorts to ensure robustness.

### Preliminary correlation between lncRNA RP11-227G15.3 levels and a specific language behavior in PSA

**Figure 5:**
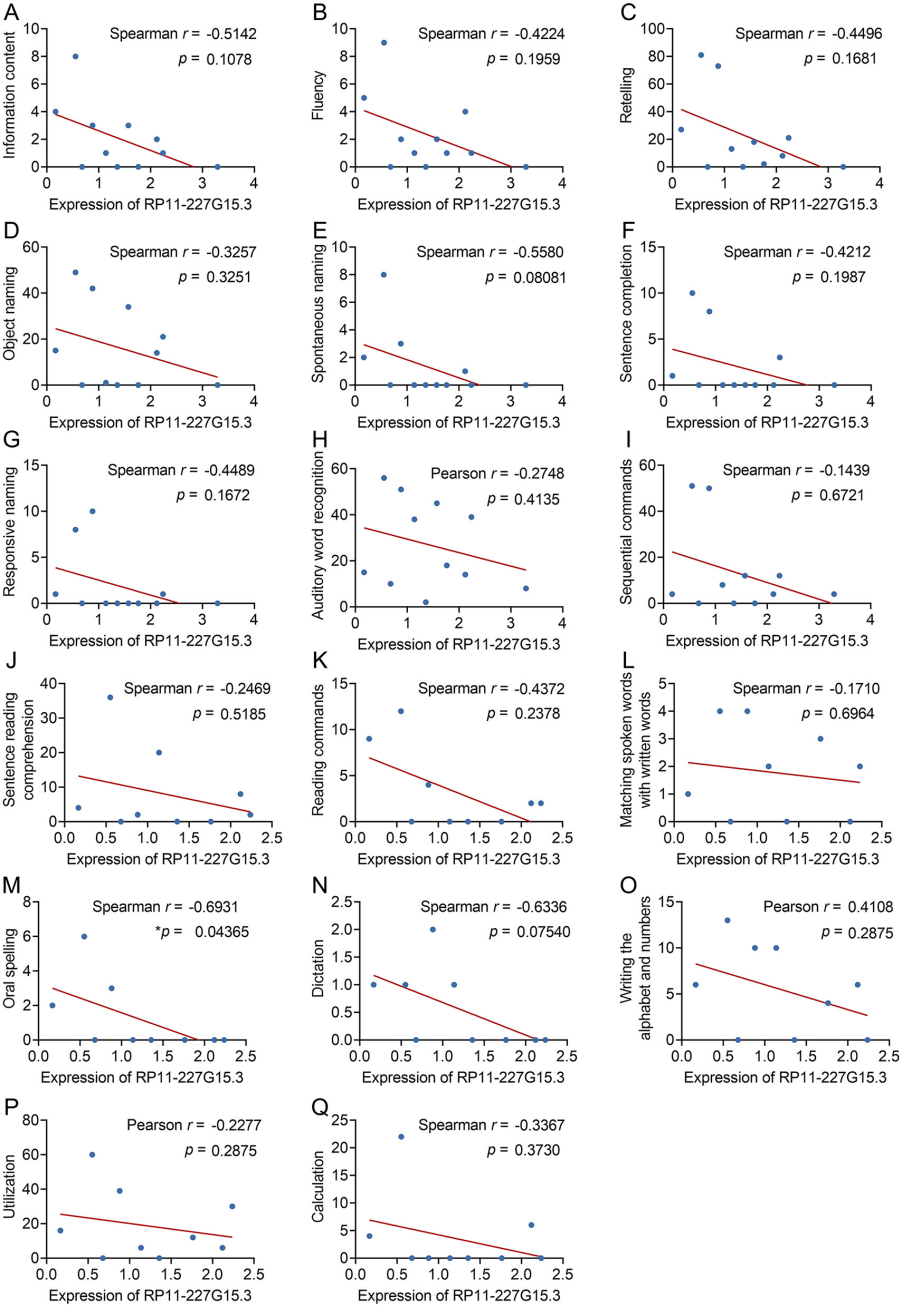
Evaluation of the correlative relationships between RP11-227G15.3 levels and language behavior test scores in patients diagnosed with PSA. **(A)** Information content (Spearman *r* = −0.5142, *p* = 0.1078); **(B)** Fluency (Spearman *r* = −0.4224, *p* = 0.1959); **(C)** Retelling (Spearman *r* = −0.4496, *p* = 0.1681); **(D)** Object naming (Spearman *r* = −0.3257, *p* = 0.3251); **(E)** Spontaneous naming (Spearman *r* = −0.5580, *p* = 0.08081); **(F)** Sentence completion (Spearman *r* = −0.4212, *p* = 0.1987); **(G)** Responsive naming (Spearman *r* = −0.4489, *p* = 0.1672); **(H)** Auditory word recognition (Pearson *r* = −0.2748, *p* = 0.4135); **(I)** Sequential commands (Spearman *r* = −0.1439, *p* = 0.6721); **(J)** Sentence reading comprehension (Spearman *r* = −0.2469, *p* = 0.5185); **(K)** Reading commands (Spearman *r* = −0.4372, *p* = 0.2378); **(L)** Matching spoken words with written words (Spearman *r* = −0.1710, *p* = 0.6964); **(M)** Oral spelling (Spearman *r* = −0.6931, *p = 0.04365 < 0.05); **(N)** Dictation (Spearman *r* = −0.6336, *p* = 0.07540); **(O)** Writing the alphabet and numbers (Pearson *r* = 0.4108, *p* = 0.2875); **(P)** Utilization (Pearson *r* = −0.2277, *p* = 0.2875); **(Q)** Calculation (Spearman *r* = −0.3367, *p* = 0.3730).

To further investigate the potential of RP11-227G15.3 as a clinical biomarker, the correlation between its expression levels and language behavior scores in PSA patients was analyzed (Supplementary File S5). Through Spearman correlation analyses (Figure 5), only oral spelling ability showed a negative correlation with RP11-227G15.3 expression (Spearman *r* = −0.6931, *p* = 0.04365). No significant associations were observed for other language measures such as information content, fluency, repetition, object naming, spontaneous naming, sentence completion, etc. Notably, this single association did not survive Bonferroni correction for multiple testing, underscoring its preliminary nature. These findings imply a potential link between RP11-227G15.3 and specific language-related deficits in PSA, rather than broad cognitive decline.

## Discussion

Here, lncRNA expression profiles were compared among PSA, PSA-T, stroke, and HC samples through complementary RNA-seq and qPCR approaches, identifying DElncRNAs and their potential functional roles in PSA through pathway enrichment analyses. After validating the expression of 6 DElncRNAs using qPCR, RP11-227G15.3 was found to be significantly upregulated in patients with PSA, with a preliminary negative correlation to oral spelling scores in a small cohort of 12 patients. It is worth noting that for this qPCR validation, we relied solely on GAPDH as the reference gene for normalization—a limitation that should be addressed in future studies by including additional reference genes. Additionally, one of the targeted lncRNAs (RP11-732A19.10) failed to be validated, which suggests there may be false positives among the initially identified differentially expressed lncRNAs, highlighting the need for rigorous validation of sequencing results. Our study design strengthens reliability by adjusting for lesion laterality and NIHSS score in statistical models ensuring aphasia status was the key distinguishing factor after accounting for differences in lesion location and stroke severity.

In GO analyses, DElncRNAs identified when comparing the PSA and stroke patient samples were enriched for terms including regulation of cell shape, immune response, regulation of transcription, and inflammatory response. These enrichments, derived from PBMC data, reflect statistical trends rather than confirmed biological mechanisms in CNS cells like microglia, as annotation biases and multiple-testing effects may influence such results. While morphological changes in microglia are observed following traumatic brain injury or in neurodegenerative disease (12), our findings do not provide evidence that PBMC-derived lncRNAs are involved in regulating glial remodeling or neural repair in the CNS; such associations remain speculative and require validation in CNS-relevant models. Microglia are the main types of immune cells activated early post stroke (13), serving as essential regulators of the stroke-related microenvironment, but PBMC-based data cannot be directly extrapolated to these central processes. DElncRNAs may be involved in regulating PSA-related inflammation, though this inference is based on enriched terms rather than direct CNS experimental evidence. RNA polymerase II-mediated transcriptional regulation provides clues to the potential roles of lncRNAs in coordinating PSA-associated gene expression, which may further shape the pathogenesis of this disease by targeting inflammation- and repair-related genes, though this requires more in-depth verification.

KEGG analyses revealed DElncRNA enrichment in key pathways related to signal transduction, metabolism, and immune activity, including the selenocompound metabolism (CTH, TXNRD2), Rap1 signaling (LCP2, SIPA1L3, THBS1, FPR1, CDC42), glycine metabolism (GAMT, CTH), NK cell-mediated cytotoxicity (LCP2, KLRD1, MICB), and VEGF signaling (BAD, CDC42). All pathways share a corrected p-value of 0.6278, indicating these are preliminary statistical associations needing further validation to confirm functional relevance. This is consistent with the exploratory nature of enrichment analyses, which are prone to annotation biases and may miss trans-regulatory interactions due to our focus on cis-targets. Selenocompound metabolism, involving CTH and TXNRD2, may relate to cytoprotection and oxidative stress responses, aligning with selenium's role in selenoprotein-mediated antioxidant defense (14) and preclinical evidence of selenocompounds reducing neuroinflammation (15). VEGF signaling, via BAD and CDC42, connects to VEGF's neuroprotective properties (16), but PBMC lncRNA associations do not confirm direct involvement in CNS angiogenesis. NK cell-mediated cytotoxicity (17), through LCP2 and KLRD1, suggests potential roles in peripheral immune modulation in PSA, while mitophagy-related pathways may relate to neuronal survival—though links to PBMC DElncRNAs require CNS model validation. These pathways offer biologically plausible connections to PSA, but their links to PBMC lncRNAs remain exploratory, not definitive mechanisms. Functionally, DElncRNAs may influence mitophagy and neuronal survival, shaping PSA pathogenesis, though this is a hypothesis derived from enrichment patterns requiring experimental confirmation. Prior studies showing lncRNAs regulate neural repair and inflammatory pathways (18–20), provide indirect support, but our GO/KEGG results are predictive and need CNS validation.

An estimated 15–42% of patients who survive an initial stroke develop PSA that impacts certain language functions such as their understanding and production of speech, writing, and reading (4). For the qPCR validation of DElncRNAs, we relied solely on GAPDH as the reference gene for normalization. This is a limitation that should be addressed in future studies by including additional reference genes. Additionally, one of the targeted lncRNAs (RP11-732A19.10) failed to be validated, which suggests there may be false positives among the initially identified differentially expressed lncRNAs and highlights the need for rigorous validation of sequencing results. The expression of RP11-227G15.3, a novel lncRNA (not annotated in NONCODE or Ensembl) located on chromosome 6q22.31 with no previously reported functional studies, showed a negative correlation specifically with oral spelling scores in 12 PSA patients. This single association (*p* = 0.04) did not survive Bonferroni correction (α = 0.0029), and no significant correlations. were observed for other language measures or combined scores. Critically, this correlation analysis was limited to only 12 PSA patients, leading to underpowered statistics that make it impossible to draw definitive conclusions about the relationship between RP11-227G15.3 and oral spelling ability. Thus, the association is purely preliminary and hypothesis-generating, rather than a robust link to broad cognitive decline. The preliminary nature of this correlation, coupled with small sample sizes in RNA-seq analyses that may underestimate true differences, emphasizes the need for large-scale validation. Resource constraints also restricted our focus to RP11-227G15.3, leaving other DElncRNAs underexplored; future studies with larger cohorts should explore these for aphasia-specific patterns.

There are multiple limitations. First, RNA-seq sample sizes were small, which may underestimate true differences. This limitation necessitates large-scale multicenter validation of the identified lncRNAs; future multi-center studies with ≥30 cases per group, strict clinical matching, technical replicates, and qPCR validation of all significant genes are needed to verify the generalizability of current findings. Second, resource constraints restricted our focus to the core lncRNA RP11-227G15.3, leaving other DElncRNAs underexplored; future studies with larger cohorts should explore these for aphasia-specific patterns. Third, PBMC-based analyses cannot reflect CNS CNS processes, requiring CNS model validation. Fourth, GO/KEGG enrichments are prone to annotation and multiple-testing biases, and focusing solely on cis-targets may miss trans-regulatory interactions, limiting functional prediction comprehensiveness. Finally, systematic studies are needed to clarify dysregulated lncRNAs' functional roles.

## Conclusion

In summary, this study is the first to systematically profile lncRNA expression in PSA patients using complementary RNA-seq and qPCR approaches, identifying DElncRNAs and their potential associations with PSA pathogenesis. Among these, RP11-227G15.3 was validated as significantly upregulated in PSA patients, with a preliminary negative correlation to oral spelling scores, supporting its status as a candidate diagnostic biomarker requiring validation. This is an exploratory pilot study; while peripheral lncRNA like RP11-227G15.3 offer accessible insights, their relevance to CNS processes and functional roles require further investigation in larger, independent cohorts and models. These findings provide a foundational framework for understanding lncRNA-mediated mechanisms in PSA, laying groundwork for developing novel diagnostic tools and therapeutic strategies, with confirmation in larger cohorts being essential before clinical application.

## Data Availability

The raw data supporting the conclusions of this article will be made available by the authors, without undue reservation.
